# Intraocular lens calculation formula developed using artificial intelligence for ultrasonic biometry

**DOI:** 10.5935/0004-2749.2024-0083

**Published:** 2025-04-07

**Authors:** Victor Antonio Kuiava, Eliseu Luiz Kuiava, Eduardo Ottobeli Chielli, Diane Marinho Ruschel, Samara Bárbara Marafon

**Affiliations:** 1 Departamento de Oftalmologia, Hospital de Clínicas de Porto Alegre, Porto Alegre, RS, Brazil; 2 Departamento de Engenharia Elétrica, Centro Universitário Internacional, São Miguel do Oeste, Santa Catarina, Brazil; 3 Departamento de Ciências pela Vida, Universidade do Oeste de Santa Catarina São Miguel do Oeste, Santa Catarina, Brazil

**Keywords:** Biometry, Intraocular lens, Cataract, Artificial intelligence

## Abstract

**Purpose:**

We developed an artificial intelligence program for calculating intraocular lenses and analyzed its accuracy rate via ultrasonic biometry. This endeavor is aimed at enhancing precision and efficacy in the selection of intraocular lenses, particularly in cases where optical biometry is unavailable.

**Methods:**

Data was collected from the *Hospital de Clínicas de Porto Alegre*, which included cases of phacoemulsification with intraocular lens implantation, in which the lens selection was based on ultrasonic biometry. The program, implemented in Python, Java, and PHP, employs the ridge regression method. Two design options were developed: a basic model, which uses only keratometry variables (K1 and K2), axial size and final target refraction in the spherical equivalent, and an advanced model, which incorporates preoperative refraction and the patient’s age. The Universal Barrett II formula was used to compare both models.

**Results:**

The sample consisted of 486 eyes from 313 patients, with 350 eyes used for program training and 136 for program validation. The spherical equivalent hit rates, with a variation of ±0.5 D, were 86% and 87.5% for the basic and advanced models, respectively, with no statistically significant difference between them. With the Barret Universal II formula, the success rate was 69%, which was significantly different from the values of the two aforementioned models (p<0.0001). The system was better for medium and long eyes but worse for short eyes (<=22.00 mm).

**Conclusion:**

The developed artificial intelligence program was superior to the Barrett formula in terms of performance, in the general context and within the subgroup of patients with longer eyes. This innovation can considerably contribute to the selection of intraocular lenses, particularly in cases where optical biometry is unavailable.

## INTRODUCTION

Cataract is one of the leading causes of reversible visual blindness worldwide, particularly in developing countries^(1^-^4)^. Cataract surgery involves the removal of the opaque lens and the implantation of an intraocular lens (IOL). A crucial element in this process is the selection of the IOL power. The selection of the dioptric power of the IOL is crucial for improving the quality of life, increasing autonomy, and reducing the cost of purchasing glasses^(5^-^7)^.

Traditionally, IOL calculation formulas have been based on static biometric measurements, such as the axial length of the eye and corneal curvature^(8)^. However, the advancements in artificial intelligence (AI) programming and the evolution of traditional IOL calculation formulas have led to considerable improvements in IOL calculation formulas through the use of optical biometry.

Optical biometers are high-cost equipment and are not always available in ophthalmic centers in developing countries, where ultrasound biometers are mostly still used to determine axial eye size^(9^,^10)^. In this context, AI has emerged as a revolutionary ally to ophthalmology, particularly for better characterization of the data of a specific population in a particular hospital. The ability of AI to analyze large datasets, learn complex patterns, and dynamically adapt to the unique characteristics of each patient has driven the development of programs^(11^,^12)^.

There are numerous examples of AI applications in medicine, including the histological identification of breast neoplasms and skin tumors^(11^,^12)^. In ophthalmology, they facilitate the identification of lesions in the fundus of the eye in retinography examinations^(13)^. Formulas that use AI have high reliability in postsurgical results^(8^,^14^,^15)^.

These programs use advanced algorithms, neural networks, and machine learning techniques to process a diverse array of biometric data, ensuring a personalized and precise calculation of IOL power specifically tailored for distinct populations in various regions worldwide^(9)^. In addition, the AI program can be designed to conduct mathematical regressions, assisting in the selection of IOLs for patients undergoing cataract surgery evaluations via ultrasound biometry.

The present study aimed to develop an AI program for calculating IOLs and to conduct an in-depth analysis of its accuracy rate via ultrasonic biometry. This endeavor is aimed at enhancing precision and efficacy in the selection of IOLs, especially in cases where optical biometry is unavailable.

## METHODS

This retrospective and observational study included 350 eyes, of which 136 were used for the validation of adult patients who underwent phacoemulsification surgery at the *Hospital de Clínicas de Porto Alegre* between March 2021 and December 2023. The data were obtained from the surgical outcomes of four cataract surgeons. The sample consisted of patients treated within this timeframe. The inclusion criteria were as follows: age over 18 years, patients undergoing phacoemulsification surgery who had preoperative assessment for IOL selection via ultrasound biometry, IOL implantation in the capsular bag, a minimum of 30 days of postoperative ophthalmological follow-up, hydrophobic three-piece IOL implantation (MA60AC, Alcon, USA), and postoperative corrected visual acuity of 20/30 (0.6) or better with refraction between 30 and 90 days postoperatively.

The exclusion criteria were missing data on keratometry, axial length, IOL used, or postoperative refraction; history of combined surgeries, such as vitrectomy, corneal transplant, or trabeculotomy; intraoperative complications, such as posterior capsule rupture, vitreous loss, and zonular weakness requiring capsular tension rings; inability to implant the IOL in the capsular bag; or postoperative complications, such as endophthalmitis, significant biometric error necessitating IOL exchange, or conditions like dry eye, blepharitis, and corneal scarring.

The patients’ medical records from March 2021 to December 2023 were reviewed, and the data collected included age, preoperative static or dynamic refraction, preoperative visual acuity, keratometry, axial length, IOL used, preoperative spherical equivalent, postoperative static or dynamic refraction, and corrected postoperative visual acuity. Axial length was measured via ultrasonic biometry (AL-100, Tomey, Japan). Furthermore, keratometry was performed in all patients (VISUREF 100, Humphrey Zeiss, Germany). The same IOL model (MA60AC, Alcon, USA) was used in all patients. Measurements were performed by an experienced operator, applying minimal pressure to avoid or minimize any impact on the axial length. The average of three high--quality measurements was calculated.

The AI program was developed using Python, Java, and PHP. In addition, a ridge regression formula was established using Python programming (Table 1).

**Table 1:** Program parameters table

Parameter	Value
alpha	0.04393970561
fit_intercept	true
max_iter	100
random_state	1234
tol	0.0001

The standard ridge regression formula with regularization in Python programming is as follows:


$$J(\beta )=\Sigma (i=1\text{ to }n)\left(y_{-}i-\left(\beta 0+\Sigma (j=1\text{ to }p)\beta j*x_{-}i\right)\right)\wedge 2+\lambda *\Sigma (j=1\text{ to }p)\beta j\wedge 2$$

**Figure 1:**
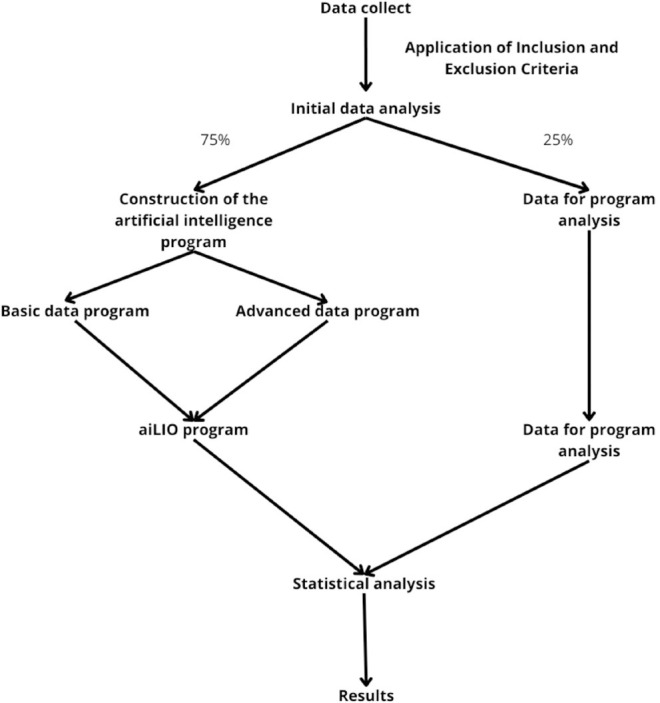
Exemplification of the project in items

The collected data were randomized, and two groups were established: one with 75% of the data for the formulation of the program’s database and the second group with 25% for statistical evaluation (Figure 1).

The formula uses two regression models: a basic model that uses keratometry variables, axial size and desired spherical equivalent and an advanced model that incorporates preoperative refraction and patient age.

Statistical analysis was conducted by comparing the values of spherical equivalents found, those described in the medical records, and those expected by the Barrett Universal II formula through the input of keratometry and axial length data. The evaluation considered errors in spherical equivalents greater than 0.5, 1, and 1.5 D.

For an interactive and practical user interface, the development of a website for utilizing the mathematical formula was proposed. The program was named aiLIO (AI IOL).

The data were tabulated in Google Sheets and were later used in the analysis using GraphPad Prism 10.1.2. Calculations were used to determine whether the data were parametric or nonparametric, with the *t*-test used for the former calculations and the Mann-Whitney U test for the latter calculations^(16)^.

The Shapiro-Wilk test was employed to evaluate the normality of the data subsets, and all variables were found to have a nonparametric distribution.

## RESULTS

This study included 486 eyes from 313 patients, with 350 eyes used for program training and 136 for program validation. The mean age of the patients was 72 years. Of them, 142 were men and 171 were women. In the database group, the mean axial was 23.19 mm, with a mean keratometry of 43.86 D, a minimum of 39.6 D, and a maximum of 48.6 D. In the test group, the mean axial length was 23.27 mm, with a mean keratometry of 43.92 D, a minimum of 40.1 D, and a maximum of 48.87 D (Table 2).

**Table 2:** Biometric data table of the two groups used

	BANK				TEST			
	**Average**	**Min.**	**Max.**	**DP**	**Average**	**Min.**	**Max.**	**DP**
KER								
K1	43.59	39	48	1.58	43.3	39.75	47.75	1.45
K2	44.16	40	49.5	1.65	44.53	41	49.75	1.98
Axial length	23.19	20.41	28.05	0.98	23.27	21.43	29.04	1.08
^*^ ≤22.00	7.7% (n=27)				5.8% ( n=8)			
^*^ ≥24.00	15.7% (n=55)				19.1% (n=26)			

Other regression models were developed using linear regression, logarithmic regression, decision tree, and elastic net regression. However, these models did not yield satisfactory results or encountered errors during the program development; thus, they were not elaborated for the program development.

**Figure 2:**
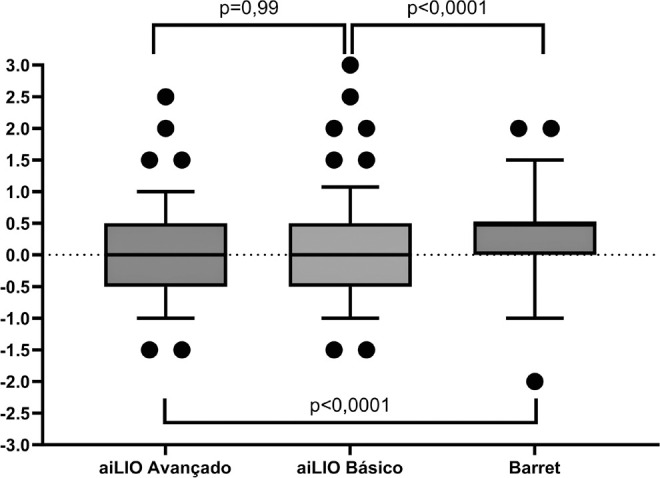
Distribution of spherical equivalent errors among the analyzed eyes with significance comparison between the programs

In the statistical analysis, the advanced aiLIO achieved an accuracy of 87.5% within +/-0.5 D, the basic aiLIO achieved 86%, whereas the Barrett Universal II formula achieved 69%. No statistically significant difference was observed between the two versions of the program (p=0.99). When the program was compared with the Barrett Universal II formula, a statistically significant difference was observed in terms of the total number of spherical equivalent errors (p<0.0001), with fewer refractive errors with the new formula (Figure 2 and Table 3).

**Table 3:** Accuracy table of programs by spherical equivalent and subgroups of long and short eyes

All data			
	**aiLIO**		
**Diopter variation**	**Advanced** **%**	**Basic** **%**	**Barret** **%**
≤0.5	87.50	86	69
≤1	95.50	94.10	93.30
≤1.5	98.50	97	99.20
>1.5	100	100	100
Total	n=136	n=136	n=136
Long eyes (≥24.00 mm)			
	aiLIO		
**Diopter variation**	**Advanced** **%**	**Basic** **%**	**Barret** **%**
≤0.5	76.90	80.70	65
≤1	96.15	96.15	96.15
≤1.5	96.15	96	100
>1.5	10	100	100
Total	n=26	n=26	n=26
Short eyes (≤22.00 mm)			
	aiLIO		
**Diopter variation**	**Advanced** **%**	**Basic** **%**	**Barret** **%**
≤0.5	50	50	50
≤1	62.50	75	75
≤1.5	87.50	87.50	100
>1.5	100	100	100
Total	n=8	n=8	n=8

**Figure 3:**
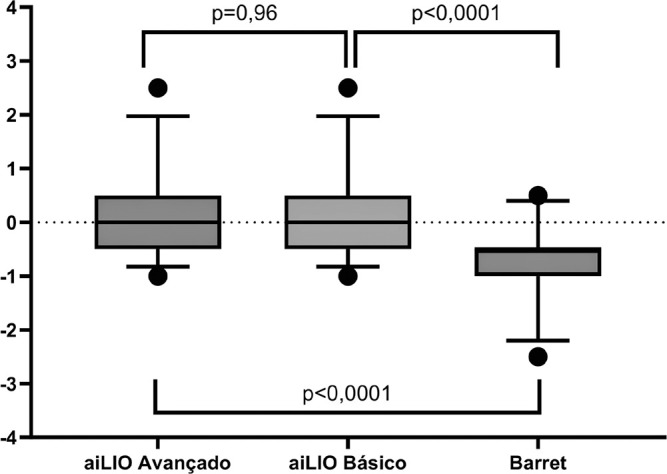
Distribution of spherical equivalent errors among the analyzed long eyes with significance comparison between the programs

In the subgroup analysis of long eyes (>=24.00 mm), no significant difference was observed between the two versions of aiLIO (p=0.96), but there was when compared with the Barrett Universal II formula. The advanced aiLIO achieved an accuracy of 76.9% (p<0.0001), the basic aiLIO achieved 80.7% (*p*<0.0001), whereas the Barrett Universal II formula achieved 65% (Figure 3).

**Figure 4:**
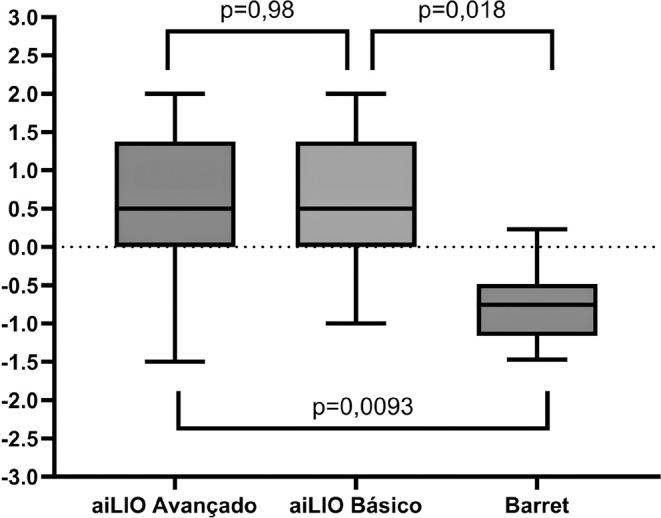
Distribution of spherical equivalent errors among the analyzed short eyes with significance comparison between the programs

In short eyes (≤22.00 mm), no difference was obser-ved between the two versions of aiLIO (p=0.98); however, the Barrett Formula was found to be better in calculating short eyes, achieving an accuracy of 50% (p<0.05) (Figure 4).

**Figure 5:**
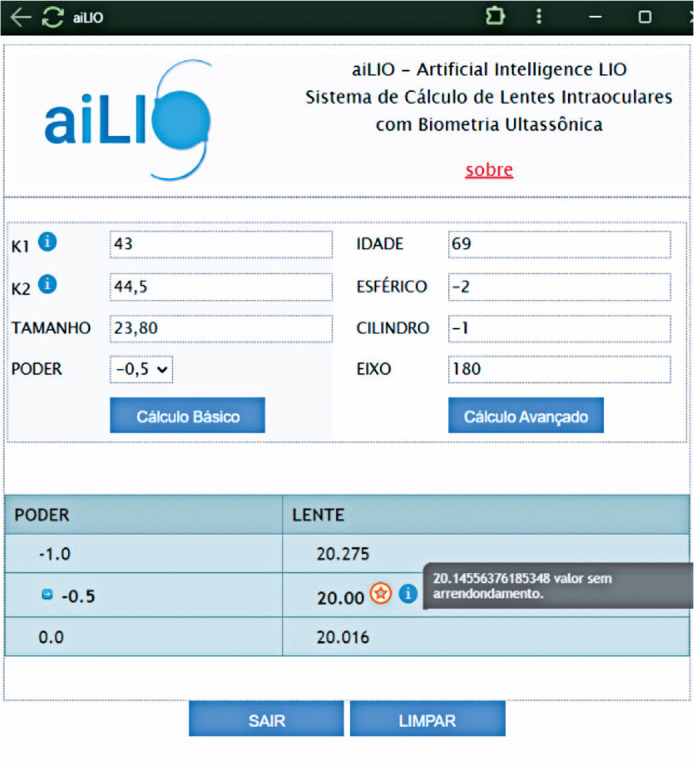
Layout of the computational program for the intraocular lens calculation (aiLIO)

Regarding the esthetic aspect of the program, an intuitive and simple layout was created using JAVA for ease of use. A system was implemented to convert “,” to “.” for calculation within the system. The postoperative refractive target values were placed in a testbed session ranging from +1.00 to -2.00, with intervals of 0.5 D. The results are presented in the lower table, where the only rounded value is the suggested lens value (Figure 5).

An example of the calculation of the dioptric power of an IOL is presented in figure 5, where a 69-year-old patient with a refraction of 2.00 1.00 at 180°, corneal curvature of 43.00 × 44.50, axial length of 23.80 mm, and a desired postoperative refractive result of -0.50, the best lens choice would be a +20.00 lens.

## DISCUSSION

The present study aimed to develop an AI program specifically designed for calculating IOLs and to conduct an in-depth analysis of its accuracy via ultrasonic biometry. This endeavor is aimed at enhancing precision and efficacy in the selection of IOLs, particularly in cases where optical biometry is unavailable. The thorough investigation sought to elucidate the potential of the AI program in providing reliable estimated and optimizing the surgical planning process in clinical settings lacking optical biometry resources, thereby establishing new paradigms of technological innovation in modern ophthalmology.

We herein developed a ridge regression program using AI. This program outperformed the Barrett formula in terms of overall performance in the general context and within the subgroup of patients with longer eyes. However, it exhibited inferiority for shorter eyes. IOL calculation in short eyes presents unique challenges in terms of the effective positioning of the IOL, measurement of the depth of the anterior chamber of the eye, and the likelihood of steeper corneas^(10^,^17)^.

Furthermore, due to the stringent criteria applied in the patient selection, the study included only a limited number of patients with extreme axial lengths, particularly those with short eyes (≤22.00 mm), totaling 35 eyes.

The simplicity of the input data in our developed formula is advantageous, considering its widespread applicability and efficacy in ophthalmology services globally^(10)^. All the values required for calculation are fundamental and readily accessible through routine ophthalmic consultations and the use of keratometry and biometry devices.

While additional parameters, such as the depth of the anterior chamber of the eye, lens thickness, white-to-white ratio, and effective lens position, are indeed valuable for IOL calculation^(10)^, their absence in the medical records posed a challenge. Consequently, these parameters were not included as mandatory data in the development process. The absence of these data was due to the retrospective nature of the study, as it relies on the accurate documentation of all values in the patients’ medical records.

Ultrasonic biometrics can be used in two different modalities: contact or immersion. Immersion is preferred because the probe of the device does not come into direct contact with the cornea, thereby minimizing the risk of indenting the cornea and underestimating the axial length of the eye.

Ultrasound and optical biometries are comparable under ideal conditions, exhibiting minimal differences in the results between the two methods^(9)^. At present, considerable efforts are directed toward the development and refinement of automated optical biometry devices so as to enhance the precision of calculating premium lenses.

This study provides a significant advantage by improving calculation accuracy for IOL services in ophthalmology settings lacking access to optical biometers and solely relying on ultrasound biometry. This is particularly crucial as it ensures that patients, even in resource-constrained settings, receive precise calculations for their cataract surgeries and other ophthalmic interventions.

In conclusion, this study developed an advanced AI program that leverages ultrasonic biometry to accurately calculate IOL power, which demonstrated considerable superiority to the Barrett Universal II formula in terms of performance, particularly for eyes with standard and long axial lengths. Despite the limitations faced with short eyes and the retrospective nature of data collection, this AI-driven program provides a robust solution for clinical settings where optical biometry is unavailable, thereby enhancing precision in surgical planning. In addition to its ability to deliver precise refractive predictions, the program’s intuitive design highlights its potential as a transformative tool in the field of ophthalmology, ensuring that even resource-constrained environments can achieve reliable outcomes in cataract surgeries and other ophthalmic procedures. This advancement not only broadens the scope of IOL calculations but also sets a precedent for future innovations integrating AI in medical technologies.
